# Supplementary material for: Small amounts of misassembly can have disproportionate effects on pangenome-based metagenomic analyses
Source: bioRxiv. 2024 Oct 13:2024.10.11.617902. Preprint. [Version 1] doi: 10.1101/2024.10.11.617902 (PMC11482961; doi:10.1101/2024.10.11.617902)
Supplement: Supplement 1 [file NIHPP2024.10.11.617902v1-supplement-1.pdf]

bioRxiv preprint doi: https://doi.org/10.1101/2024.10.11.617902; this version posted October 13, 2024. The copyright holder for this preprint (which was not certified by peer review) is the author/funder, who has granted bioRxiv a license to display the preprint in perpetuity. It is made available under aCC-BY 4.0 International license.

# Supplementary Information

**Supplementary Table 1:** An expanded version of Table 1 that also includes the UHGP-90 ID each gene maps to, and the total number of genomes it was found in.

**Supplementary Figure 1:** Density plots showing CheckM-estimated percent contamination for genomes containing genes identified as contaminants vs. all other genomes in the same species.

**Supplementary Figure 2:** Receiver-operator characteristic (ROC) curves showing how well the correlation test ("family" and "species"), EggNOG-predicted taxonomic ranges ("EggNOG"), and statistical significance ("FDR") predict contamination, as ascertained via BLAST. For the correlation test, we report results for the top-ranked match at the family level or below (red, "family"), as well as the top-ranked match at the species level only (purple, "species").

**Supplementary Figure 3:** Heat map showing MIDAS2-estimated copy numbers of flagellar genes in the *L. eligens* pangenome. Genes are clustered using Pearson correlation and subjects are clustered using Euclidean distance. Genes significantly associated with cirrhosis (Fisher's test, adjusted p-value $\leq 0.05$) are marked in black. The left-side colors show PAM clusters of genes that were significantly associated with cirrhosis (red), controls (blue), or neither (white); clusters with too few genes to perform association tests are colored gray. The right-side "enrichment" colors summarize differences in gene copy number between cases and controls (t-statistic, mean of 500 bootstrap samples; red is enriched in cirrhosis, while blue is enriched in cases).
